# Late-Life Alcohol Exposure Does Not Exacerbate Age-Dependent Reductions in Mouse Spatial Memory and Brain TFEB Activity

**DOI:** 10.3390/biom14121537

**Published:** 2024-11-30

**Authors:** Hao Chen, Kaitlyn Hinz, Chen Zhang, Yssa Rodriguez, Sha Neisha Williams, Mengwei Niu, Xiaowen Ma, Xiaojuan Chao, Alexandria L. Frazier, Kenneth E. McCarson, Xiaowan Wang, Zheyun Peng, Wanqing Liu, Hong-Min Ni, Jianhua Zhang, Russell H. Swerdlow, Wen-Xing Ding

**Affiliations:** 1 Department of Pharmacology, Toxicology and Therapeutics, The University of Kansas Medical Center, Kansas City, KS 66160, USA; hac4011@med.cornell.edu (H.C.); kgreenwood@kumc.edu (K.H.); czhang10@kumc.edu (C.Z.); yrod889@gmail.com (Y.R.); swilliams30@kumc.edu (S.N.W.); mniu@kumc.edu (M.N.); xiaowen.ma@newcastle.ac.uk (X.M.); chaoxj3@mail.sysu.edu.cn (X.C.); kmccarson@kumc.edu (K.E.M.); hni@kumc.edu (H.-M.N.); 2 R.L. Smith IDDRC Rodent Behavior Facility, Disease Model and Assessment Services, The University of Kansas Medical Center, Kansas City, KS 66160, USA; afrazier10@kumc.edu; 3 Department of Neurology, The University of Kansas Medical Center, Kansas City, KS 66160, USA; xwang9@kumc.edu (X.W.); rswerdlow@kumc.edu (R.H.S.); 4 Department of Pharmaceutical Sciences, Eugene Applebaum College of Pharmacy and Health Sciences, Detroit, MI 48201, USA; gp5053@wayne.edu (Z.P.); wliu@wayne.edu (W.L.); 5 Department of Pathology, Division of Molecular Cellular Pathology, University of Alabama at Birmingham, 901 19th Street South, Birmingham, AL 35294, USA; jianhuazhang@uabmc.edu; 6 Department of Internal Medicine, The University of Kansas Medical Center, Kansas City, KS 66160, USA

**Keywords:** autophagy, lipid, lysosome, mitochondria, tau aggregation

## Abstract

Alcohol consumption is believed to affect Alzheimer’s disease (AD) risk, but the contributing mechanisms are not well understood. A potential mediator of the proposed alcohol-AD connection is autophagy, a degradation pathway that maintains organelle and protein homeostasis. Autophagy is regulated through the activity of Transcription factor EB (TFEB), which promotes lysosome and autophagy-related gene expression. The purpose of this study is to explore whether chronic alcohol consumption worsens the age-related decline in TFEB-mediated lysosomal biogenesis in the brain and exacerbates cognitive decline associated with aging. To explore the effect of alcohol on brain TFEB and autophagy, we exposed young (3-month-old) and aged (23-month-old) mice to two alcohol-feeding paradigms and assessed biochemical, transcriptome, histology, and behavioral endpoints. In young mice, alcohol decreased hippocampal nuclear TFEB staining but increased SQSTM1/p62, LC3-II, ubiquitinated proteins, and phosphorylated Tau. Hippocampal TFEB activity was lower in aged mice than it was in young mice, and Gao-binge alcohol feeding did not worsen the age-related reduction in TFEB activity. Morris Water and Barnes Maze spatial memory tasks were used to characterize the effects of aging and chronic alcohol exposure (mice fed alcohol for 4 weeks). The aged mice showed worse spatial memory acquisition in both tests. Alcohol feeding slightly impaired spatial memory in the young mice, but had little effect or even slightly improved spatial memory acquisition in the aged mice. In conclusion, aging produces greater reductions in brain autophagy flux and impairment of spatial memory than alcohol consumption.

## 1. Introduction

Alzheimer’s disease (AD) is a prevalent form of age-dependent progressive dementia that affects more than 4% of individuals aged 65 years and above. The primary pathological characteristics of AD are the buildup of extracellular β-amyloid plaques and intracellular fibrillary tangles composed of aggregated hyperphosphorylated tau [1]. In addition to the accumulation of protein aggregates, mitochondrial dysfunction also plays a critical role in the pathogenesis of AD and perhaps some AD-related disorders (ADRD) [2,3]. Extensive clinical studies strongly support a link between alcohol use disorders with AD and ADRD [4,5]. Heavy alcohol drinkers have impaired cognitive function even in the absence of a dementia diagnosis. Individuals with alcohol use disorders also have a two-fold greater risk of developing AD versus the general population [6]. In a study involving a cohort of 360 patients with early AD who were followed biannually for up to 19 years, it was observed that heavy drinkers—defined as those consuming eight or more alcoholic drinks per week—experienced a more rapid cognitive decline. In contrast, there was no significant difference in cognitive decline between mild to moderate drinkers and those who abstained from alcohol [7]. However, some epidemiology studies suggest that moderate drinking is beneficial and decreases the risk of dementia [8,9]. These divergent findings may reflect inconsistencies in alcohol and AD measurement parameters. However, the molecular mechanisms underlying the effects of alcohol on brain function and the pathogenesis of AD remain largely unknown.

Autophagy is a highly conserved lysosomal degradation pathway that is activated in response to stress, such as deprivation of nutrients or growth factors, as a survival mechanism [10,11]. Autophagy can help remove excess protein aggregates (aggrephagy) or damaged organelles such as mitochondria (mitophagy) to protect against cell death [12,13,14,15]. The lysosome is the terminal component of autophagy and contains more than 50 acid hydrolases. The transcriptional regulation of genes that facilitate lysosome biogenesis and autophagy plays a critical role in autophagy [16]. Transcription factor EB (TFEB) is a basic helix-loop-helix leucine zipper transcription factor belonging to the coordinated lysosomal expression and regulation (CLEAR) gene network [16]. TFEB is a master regulator for transcription of lysosome biogenesis and autophagy genes [17,18]. In response to an increased need for autophagic degradation, TFEB coordinates an efficient transcriptional program that upregulates the expression of genes that are responsible for both the early (autophagosome formation) and late (lysosome biogenesis) phases of autophagy. Deletion of *hlh-30*, an orthologue of TFEB in *C. elegans*, resulted in reduced autophagosome formation and a decrease in lysosome-mediated degradation of autophagic substrates, leading to a shortened lifespan of *C. elegans* [19]. We and others have demonstrated that alcohol consumption impairs TFEB-mediated lysosomal biogenesis and autophagic flux in the liver and pancreas, which promotes alcoholic hepatitis and pancreatitis [20,21,22]. Whether and how alcohol affects TFEB in the brain, and especially in the hippocampi that are affected early in AD, has not been studied.

In addition to alcohol abuse, increased age is a well-known risk factor for AD. Both autophagy and lysosomal functions decline with aging [23,24]. It has been demonstrated that the brains of individuals with AD have impaired autophagy. This results in excessive accumulation of immature autophagosomes, which is caused by defects in the fusion of autophagosomes with lysosomes or hindered retrograde traffic of autophagosomes towards the neuronal cell body [25]. In AD, a buildup of immature autophagosomes can cause inadequate autophagy, leading to the accumulation of tau and β-amyloid plaques. A decrease in nuclear TFEB levels has been observed in the subcellular fractionation analysis of AD patient brains. There is a strong inverse correlation between hippocampal nuclear TFEB levels and the severity of AD pathology [26]. Moreover, decreased TFEB function has been observed in AD patient lymphocytes and monocytes, which may migrate to damaged *central nervous system* (CNS) regions and regulate AD progression [27]. Furthermore, expression levels of the CLEAR gene network also decreased in cultured fibroblasts from AD patients and iPSC-derived human AD neurons [28]. More importantly, overexpression of TFEB promotes the clearance of aberrant tau proteins in the rTg4510 and P301S mouse models of tauopathy [29,30]. SIRT1, an NAD+-dependent deacetylase, deacetylates TFEB at lysine 116 and this induces TFEB-mediated lysosomal biogenesis that promotes the degradation of fibrillar Aβ by microglia [31]. More recently, the curcumin analog C1 was shown to activate TFEB and increase lysosome biogenesis, which in turn decreased beta-amyloid precursor protein and Tau pathology in three AD animal models (5XFAD, P301S, and 3XTg-AD) [32].

In this study, we used chronic plus and binge alcohol models to explore how alcohol affects autophagy-lysosome-related pathways in mouse brains. We also used a chronic alcohol feeding model in young and aged mice to investigate whether age and alcohol affected cognitive performance in memory acquisition tasks. Our results show that aging and alcohol impair TFEB signaling in the brains of young mice, and aging, but not alcohol consumption resulted in decrements in cognitive performance of old mice.

## 2. Materials and Methods

### 2.1. Animal Experiments

Male 3- and 23-month-old C57BL/6N mice were obtained from Charles River Laboratories (Wilmington, MA, USA) and housed at the AAALAC-accredited facility at KUMC for at least 1 week on a regular chow diet for acclimation. All mice were specific pathogen-free (SPF) and received human care in a barrier rodent facility under standard experimental conditions. Animal studies were approved and performed under the Institutional Animal Care and Use Committee (IACUC) at the University of Kansas Medical Center (Kansas City, KS, USA).

Subsequently, for the Gao-binge alcohol model, mice were acclimated to the Lieber-DeCarli liquid control diet (F1259SP, Bio-Serv, Flemington, NJ, USA) for 5 days followed by further feeding with the liquid control or ethanol diet (F1258SP, Bio-Serv, 5% ethanol) for 10 days. The volume of the control diet given to mice was matched to the volume of ethanol diet consumed. On the last day of feeding, mice were further given 5 g/kg ethanol or 9 g/kg maltose dextran and sacrificed 8 h later. On the last day of feeding, mice were euthanized 8 h after the gavage. Brain tissues and blood samples were collected. Left-hemi brains were collected for western blotting and right-hemi brains were obtained for cryosection, hematoxylin, and eosin (H&E) staining as well as confocal microscopy.

Prior to behavioral testing, mice were acclimated to the Lieber-DeCarli liquid control diet) for 5 days followed by further feeding with the liquid control or ethanol diet (5% ethanol) for 4 weeks. After the last day of alcohol and liquid control diet feeding, mice were returned to the chow diet for recovery of 3 days followed by either Morris Water Maze or Barnes Maze testing. For the Morris Water Maze test, a 3-months-old (N = 4) fed with a control diet (Control), 3-months-old (N = 4) fed with an ethanol diet (EtOH), 23-months-old (N = 4) fed with a control diet (Control), 23-months-old (N = 6) fed with the ethanol diet (EtOH). For the Barnes Maze test, 3-months-old (N = 5) fed with the control diet (Control), 3-months-old (N = 5) fed with the ethanol diet (EtOH), 23-months-old (N = 5) fed with the control diet (Control), and 23-months-old (N = 4) fed with the ethanol diet (EtOH) were used.

### 2.2. Mouse Behavioral Testing

The Morris Water Maze test was used to assess spatial memory acquisition and retention using well-defined protocols [33,34]. Mice acclimated to the testing environment for at least 1 hour before testing. Mice were randomized by diet treatment group, to which the experimenter was blinded during testing. In brief, mice learn to find a hidden platform (20 cm diameter) in an open swimming pool (200 cm diameter) filled with 21 °C water, which was made opaque with nontoxic poster paint. A digital camera suspended above the maze recorded the animal’s position during the test. Four trials were performed each day for 5 days. Each trial started at a different position (N, E, SE, NW) while the platform was kept in a fixed location (NE). Each trial lasted 60 s, followed by 30 s during which mice were allowed to remain on the platform to strengthen their memory of platform location. After 5 days of memory acquisition trials, mice were subjected to a probe (retention) trial in which the platform was removed. The latency to reaching the platform, the time they spent in the target quadrant, and the number of times they passed the previous platform location were analyzed by using the animal tracker plugin of ImageJ Version 2.9.0 (National Institutes of Health).

A Barnes Maze testing protocol was adapted from previous descriptions [35], using a Barnes Maze table 1.0 m in diameter with twenty 5.0 cm escape holes in an equidistant array around the edge of the table. Four fixed objects were suspended beyond the edges of the table to provide external visual cues. Only one of the twenty escape holes contained a darkened goal box underneath, and the same hole was used as the goal across the 5 days of testing. The memory acquisition protocol consisted of one acclimation session followed by five training sessions. Observers were blind to individual subject treatments. The mice were initially acclimated to the goal maze by placing them within a goal box located outside of the Barnes Maze table for 2 min. Goal box acclimation preceded the first training session by at least 1 day. During the first training session, the mice were placed in the center of the Barnes Maze platform covered by an inverted paper container for approximately 20 s, and then released to find the goal box. If the mouse failed to find the goal within 3 min, the test trial was terminated, then the mouse was gently guided to the goal and kept there for one minute before being returned to the home cage. This training was repeated for five consecutive days, with four trials occurring each day per mouse and an inter-trial interval of no less than 5 min. The trials were recorded using an overhead digital camera and subsequently analyzed using EthoVision XT (Version 17.0.1630, Noldus Information Technology, Wageningen, The Netherlands). Data collected included latency to the goal box, total distance and velocity traveled, the daily average for latency to the goal box and distance traveled, and target visit errors to opposite/adjacent/and goal locations within the Barnes arena.

### 2.3. RNAseq Data Analysis

Total RNA was extracted from mouse hippocampi using TRIzol reagent (15596-026; Ambion, ThermoFisher Scientific, Waltham, MA, USA) and was reverse-transcribed into cDNA using RevertAid Reverse Transcriptase (EP0442; Fermentas, ThermoFisher Scientific). A detailed RNA sequencing analysis was performed as described previously [36]. Briefly, the raw data for RNA sequencing is stored in the fastq.gz file. The data quality was accessed by the FASTQC software (version 0.11.8). The bases with a per-base sequence quality lower than 30 were removed later in the QC process. Adaptors were also removed using Trimmomatic (version 0.39) software, if any. The adaptor-removed files were mapped to the mouse genome (grcm38) with HISAT2 software (version 2.0.1). The total counts for each read were accessed by HISAT2 using Mus_musculus.GRCm38.100.gtf file. The count files were then imported into Rstudio (version 2023.3.0.386, PBC, Boston, MA, USA) for downstream analysis using the DESeq package (version 3.19). The count number for each gene was log10 transformed and divided by the size factor for normalization by using the package before performing principal component analysis (PCA). Quantification of gene expression was generated with HTSeq-counts v0.6.0. Significant differentially expressed genes (DEGs) were identified with the R package DESeq2. Multiple comparisons were taken into account by converting *p* values to Benjamini−Hochberg’s false discovery rate (FDR).

Volcano plots were prepared with R software Version 4.2.0 (https://www.r-project.org/). Pathway analysis was performed using the online tool ShinyGO v0.741. We analyzed the enrichment of up-and down-regulated genes among each EtOH group as compared to the control group. Genes with a log2 fold change >1 or <−1 with an FDR-corrected *p*-value < 0.05 were used to perform the analysis. Heatmap analysis based on Gene Ontology (GO) and Kyoto Encyclopedia of Genes and Genomes (KEGG) enrichment analyses and circos plots of the DEGs were analyzed using Metascape (http://metascape.org). In the differentiation analysis, the gene expression in the experimental group was compared to that of the control group. The heatmaps were generated by the pheatmap package (version 1.0.12) using the normalized count file and the data were scaled by row.

### 2.4. Western Blot Analysis

Total brain hippocampi or cortex lysates were prepared in radioimmunoprecipitation assay buffer [1% NP-40, 0.5% sodium deoxycholate, and 0.1% sodium dodecyl (lauryl) sulfate]. Protein (20–30 µg) was separated by a 12% SDS- PAGE gel and then transferred to a polyvinylidene difluoride (PVDF) membrane. After blocking in TBS buffer (20 mM Tris-HCl, 150 mM sodium chloride) containing 5% (wt/vol) nonfat dry milk for 1 h at room temperature, the membranes were then probed with primary and secondary antibodies, followed by development with Super Signal West Pico chemiluminescent substrate (34080; Life Technologies, Carlsbad, CA, USA). Data analysis was performed by Image Lab 6.1 (Bio-Rad, Hercules, CA, USA). The following primary antibodies were used: mouse anti-p62 (Abnova, Taipei City, Taiwan H00008878-M01, 1:10,000), mouse anti-phospho-Tau (ser396) (Cell Signaling Technology, Danvers, MA, USA, 9632S, 1:3000), rabbit anti-phospho-Tau (ser404) (Cell Signaling, 20194S, 1:2000), mouse anti-total-Tau (Cell Signaling, 4019S, 1:3000), rabbit anti-TFEB (Bethyl, Montgomery, TX, USA, A303-673A, 1:1000), mouse anti-β-actin (Sigma-Aldrich, St. Louis, MO, USA, A5411, 1:10,000), mouse anti-PSD95 (Abcam, Cambridge, UK, ab2723, 1:1000), rabbit anti-Synapsin 1 (Millipore-Sigma, Burlington, MA, USA, AB1543, 1:1000), mouse anti-ubiquitin (Santa Cruz Biotechnology, Dallas, TX, USA, sc-8017, 1:3000), and rabbit anti-GAPDH (Cell Signaling, 2118, 1:5000). The following secondary antibodies were used: HRP-goat anti-Mouse IgG (Jackson ImmunoResearch, West Grove, PA, USA, 115-035-146, 1:2000) and HRP-goat anti-Rabbit IgG (Jackson ImmunoResearch, West Grove, PA, USA, 111-035-144, 1:2000). Original western blots can be found at Supplementary Materials. 

### 2.5. Histology and Immunohistochemistry (IHC) Staining

Fresh brain tissues were fixed in 10% neutral-buffered formalin overnight, transferred to 70% ethanol for at least 24 h, embedded in paraffin wax, and cut into 5 μm sections. Paraffin-embedded brain sections were further stained with hematoxylin and eosin (H & E) using a protocol described previously [37].

Additionally, immunostaining with indicated antibodies was performed as previously described. Briefly, paraffin-embedded tissue sections were incubated with primary antibody at 4 °C overnight after deparaffinization and heat-induced antigen retrieval in citrate buffer. Sections were then washed and incubated with secondary antibody for 1h at 37 °C and developed with ImmPACT Nova RED HRP substrate (Vector Labs, Newark, CA, USA, SK-4805). Tissues were counterstained with hematoxylin. The following antibodies were used: rabbit anti-TFEB (Bethyl, Montgomery, TX, USA, A303-673A, 1:1000) and HRP Horse Anti-Rabbit IgG (Vector, MP7401, one drop). Images were acquired under a Nikon Eclipse Ni microscope with a Nikon Digital SIGHT DS-U3 camera (Melville, NY, USA).

### 2.6. Electron Microscopy Analysis

Male mice aged 3 and 23 months were fed with the Gao-binge alcohol model. Their hippocampal tissues were fixed with 2% glutaraldehyde in 0.1 M phosphate buffer (pH 7.4), then treated with 1% OsO4. The tissues were embedded in hardened resin and sectioned to about 60–100 nm. These thin sections were then stained with uranyl acetate and lead citrate before being mounted on specimen grids. Digital images were taken with a JEM 1016CX electron microscope (JEOL, Peabody, MA, USA).

### 2.7. Immunofluorescence Staining

Brain-frozen sections from 3- and 24-month-old mice with or without ethanol treatment were used to perform immunostaining. Antigen retrieval was done by boiling the samples in a citrated buffer for 15 min, followed by blocking with 5% BSA/PBS and 0.5% triton-X for 1 h at room temperature (RT). Slides were mounted in PermaFluor Aqueous Mounting Medium (Thermo Scientific, TA-030-FM) followed by confocal microscopy. The following primary antibodies were used: mouse anti-total-Tau (Cell Signaling, 4019S, 1:3000) and rabbit anti-TFEB (Bethyl, A303-673A, 1:300). The following secondary antibodies were used: Alex488 goat anti-mouse (Jackson, 115-545-146, 1:600) and Alex594 goat anti-rabbit (Jackson, 111-505-144, 1:600). Fluorescence images were acquired under Nikon A1R confocal microscope (Melville, NY, USA).

### 2.8. Statistical Analysis

Statistical comparisons were performed using GraphPad Prism 8 software (https://www.graphpad.com/scientific-software/prism/, accessed on 20 September 2024). No randomization was performed, but data collection and analysis were conducted blindly to the conditions of the experiments by researchers who were blinded to group assignment. Two-way ANOVA followed by Bonferroni post hoc analysis or unpaired Student’s *t*-test followed by Bonferroni post hoc analysis were applied for data analysis. The numbers of replicates and *p*-values are stated in each figure legend. All data are expressed as mean ± SEM. Significance is indicated by the following symbols: * *p* < 0.05, ** *p* < 0.01, *** *p* < 0.001.

## 3. Results

### 3.1. Aging and Chronic Plus Binge EtOH (Gao-Binge) Feeding Impair TFEB-Mediated Autophagy in Mouse Brains

**Figure 1:**
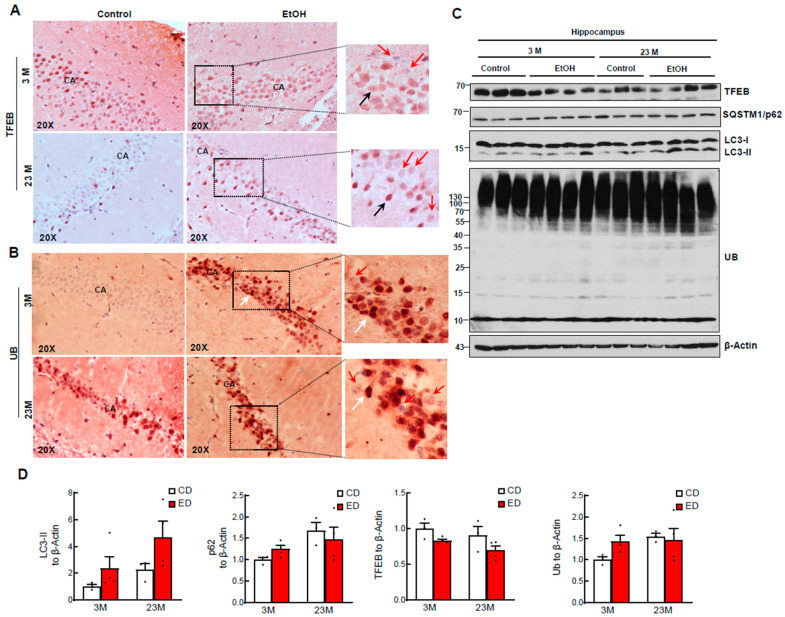
Aging and chronic plus binge EtOH (Gao-binge) feeding impair TFEB-mediated autophagy in mouse brains. Male 3-month-old young mice (3M) and 23-month-old aged mice (23M) were subjected to Gao-binge alcohol feeding. Representative images of immunohistochemistry staining for TFEB (**A**) and ubiquitin (UB) (**B**) are shown. Black and white arrows denote positive TFEB and UB stained cells whereas red arrows denote decreased TFEB and UB staining in hippocampi CA1 (Cornu Ammonis) region. (**C**) Total lysates from the hippocampus were subjected to western blot analysis. (**D**) Densitometry analysis from (**C**). Data are presented as mean ± SEM (N = 3–4). CD: Control diet, ED: ethanol diet with a binge.

We first determined the changes of TFEB and several autophagy-related markers in alcohol-fed young and aged mouse hippocampi. LC3-II is a specific marker of autophagosomes although it is degraded by lysosomal proteases and/or deconjugated by ATG4B in autolysosomes. SQSTM1/p62 is an autophagy substrate protein together with ubiquitinated misfolded proteins that are degraded in autolysosomes during the autophagy process [38]. Results from IHC staining revealed that alcohol feeding decreased nuclear TFEB and increased ubiquitin staining in young mouse hippocampi (Figure 1A,B). Aged mice had decreased nuclear TFEB and increased ubiquitin staining regardless of alcohol feeding (Figure 1A,B). Consistent with the IHC results, western blot analysis showed decreased TFEB but increased levels of ubiquitinated proteins in alcohol-fed young mouse hippocampi, suggesting alcohol impairs hippocampi autophagy in young mice. The smear pattern of ubiquitinated proteins suggests that multiple brain hippocampi proteins are ubiquitinated. Aged mice had decreased nuclear TFEB and increased ubiquitin staining, and alcohol feeding did not further alter this finding (Figure 1C). Gao-binge alcohol feeding did not appreciably alter p62 but slightly increased LC3-II levels in young mouse hippocampi. The p62 levels were also comparable between young and aged mice regardless of alcohol feeding. However, the levels of LC3-II increased in control diet-fed aged mouse hippocampi and further increased with alcohol feeding (Figure 1C,D). Increased levels of LC3-II in alcohol-fed aged mice could be attributed to either enhanced autophagosome formation or reduced lysosomal degradation, which requires further investigation in the future.

**Figure 2:**
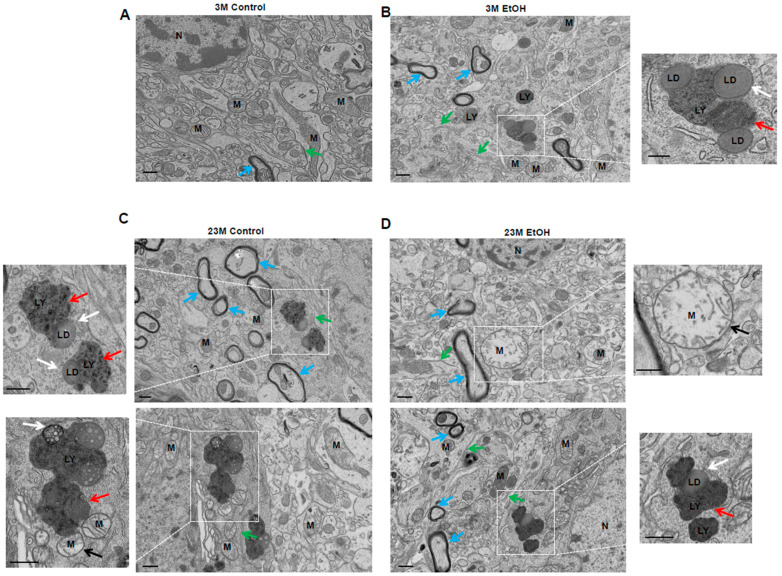
Electron microscopy analysis of the ultrastructure of Gao-binge alcohol-fed young and aged mouse hippocampi. Male 3-month-old young mice (3M) and 23-month-old aged mice (23M) were subjected to Gao-binge alcohol feeding. Representative EM images of mouse hippocampi from 3M control (**A**), ED (**B**), 23M control (**C**), and 23M ED (**D**) mice. The right panels are enlarged images from the boxed area in (**B**,**D**). The left panels are enlarged images from (**C**). Red arrows denote lysosomes (LY); white arrows denote lipid droplets (LD) in lysosomes; black arrows denote damaged mitochondria; blue arrows denote myelinated axon with lamellar sheath; and green arrows denote endoplasmic reticulum. LY: lysosome, LD: lipid droplet, M: mitochondria, N: nucleus. Bar: 500 nm.

Accumulating Aβ fibrils and lipofuscin are often found in late endosomes/lysosomes of AD patient brains [39]. EM ultrastructure analysis revealed an accumulation of abnormal lysosome structures enveloping lipid droplets (LD) and other electron-dense materials as well as the inclusion of body-like structures reminiscent of lipofuscin granules and Aβ fibril-like structures in alcohol-fed but not control diet-fed young mouse hippocampi (Figure 2A,B). Aged mice had increased lysosomes containing lipofuscin regardless of alcohol feeding (Figure 2C,D). Moreover, swollen mitochondria with abnormal cristae were readily detected in aged mouse hippocampi regardless of alcohol feeding (Figure 2C,D). Together, these results suggest that aging and chronic plus binge alcohol feeding impair TFEB-mediated autophagy and lead to the accumulation of abnormal lysosome structures containing lipofuscin and Aβ fibril-like structures. Aging alone showed significant defects in brain autophagy. Moreover, the combined effects of aging and alcohol did not reveal any additive or synergistic interaction.

### 3.2. Transcriptomic Analysis of Hippocampi from Chronic Plus Binge EtOH (Gao-Binge) Fed Young and Aged Mice

**Figure 3:**
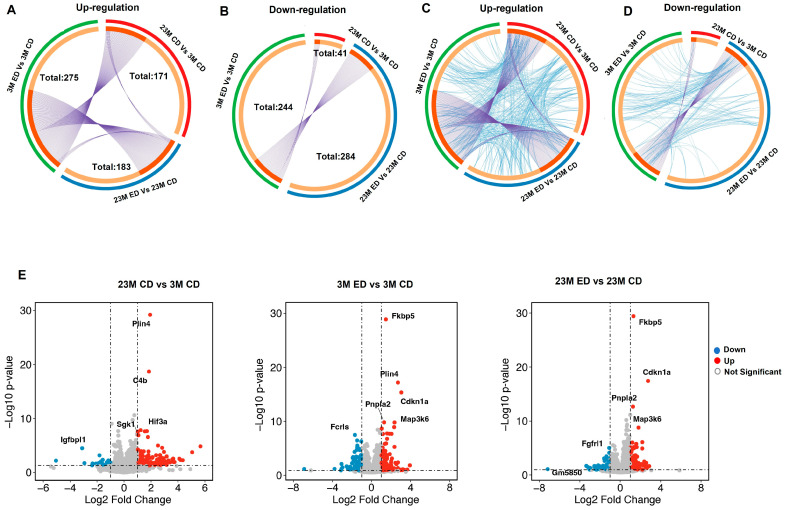
Transcriptomic analysis of Gao-binge alcohol-fed young and aged mouse hippocampi. Male 3-month-old young mice (3M) and 23-month-old aged mice (23M) were subjected to Gao-binge alcohol feeding. mRNAs were extracted from the mouse hippocampi after the feeding and subjected to RNAseq analysis. (**A**–**D**) Circos plot analysis from the RNAseq data set. Red: 23M CD (control diet) vs. 3M CD; blue: 23M ED vs. 23M CD; green: 3M ED vs. 3M CD. The inner circle represents gene lists, where hits are arranged along the arc. Purple curves link identical genes. Genes that hit multiple lists are colored in dark orange, and genes unique to a list are shown in light orange. Each arc represents a distinct group of genes, color-coded to align with the corresponding experimental conditions, while the interconnecting chords depict shared ontology terms, including the shared term level, where blue curves link genes that belong to the same enriched ontology term. The thickness of each chord is proportional to the number of genes that contribute to the common functional annotation analysis (**C**) for up-regulated genes, and (**D**) for down-regulated genes. (**E**) Volcano plots illustrating differential gene expression across experimental conditions. The x-axis represents the log2 fold change, and the y-axis represents the log10 *p*-value, indicating the magnitude and statistical significance of gene expression changes, respectively. Upregulated genes are marked in red, downregulated genes in blue, and non-significant changes in gray. Key genes with a fold change greater than 2 and a *p*-value less than 0.05 are labeled.

As shown in the Circos plot, 171 genes were significantly upregulated. In contrast, only 41 genes were significantly downregulated in control diet-fed aged mice compared with control diet-fed young mice (Figure 3A,B). Alcohol-fed young mice had 275 genes upregulated, but 244 genes were downregulated compared with control diet-fed young mice. In contrast, alcohol-fed aged mice had 183 upregulated genes, but 284 genes were downregulated compared with control diet-fed aged mice (Figure 3A,B). Among the upregulated genes, 169 genes were exclusively upregulated in control diet-fed aged mice compared with control diet-fed young mice, 115 genes in alcohol-fed aged mice compared with control diet-fed aged mice, and 148 genes in alcohol-fed young mice compared with control diet-fed young mice, respectively (Figure 3A). Among the commonly upregulated genes, 59 genes were found between “23M CD vs. 3M CD” and “3M ED vs. 3M CD”, suggesting these genes may be induced either by aging alone or by alcohol in young mice. In contrast, 62 genes were shared between “23M ED vs. 23M CD” and “3M ED vs. 3M CD”, suggesting these genes were mainly affected by alcohol independent of age (Figure 3A). 

Among the commonly downregulated genes, 33 genes were found exclusively downregulated in control diet-fed aged mice compared with control diet-fed young mice, 250 genes in alcohol-fed aged mice compared with control diet-fed aged mice, and 202 genes in alcohol-fed young mice compared with control diet-fed young mice, respectively (Figure 3B). Notably, the intersection comparison of “23M CD vs. 3M CD” and “23M ED vs. 23M CD” yielded eight commonly downregulated genes (*Cenpf, Gm13690, Gm26526, Gm3756, Gm43339, Gm6170, Rpsa-ps10*, and *Top2a*) with most of them regulating cell proliferation or pseudogenes with unclear functions. The gene expression intersection among these groups indicates a potential overlap in regulatory mechanisms in alcohol and aging although only in a small subset of genes. However, there were 34 common genes downregulated between “23M ED vs. 23M CD” and “3M ED vs. 3M CD”, suggesting a subset of downregulated genes were affected by alcohol independent of age (Figure 3B). Interestingly, there were no shared downregulated genes between the “23M CD vs. 3M CD” and “23M ED vs. 23M CD” groups (Figure 3B), demonstrating no overlap of downregulated genes between alcohol and aging.

In our next comprehensive analysis, we further refined gene set overlaps by incorporating functional annotations, revealing intricate networks of shared biological processes and pathways. Figure 3C,D present a chord diagram that illustrates the functional interconnections between the genes either upregulated or downregulated in the three experimental comparison groups (23M CD vs. 3M CD, 23M ED vs. 23M CD, and 3M ED vs. 3M CD). The diagram highlighted enriched ontology terms, which refer to categories or pathways associated with gene functions. These specifically include those with fewer than 100 genes to maintain specificity, thus excluding overly general annotations. Each arc represents a distinct group of genes, color-coded to align with the corresponding experimental conditions, while the interconnecting chords depict shared ontology terms. The thickness of each chord is proportional to the number of genes that contribute to the common functional annotation, highlighting the most significantly enriched pathways. Compared to the down-regulated gene network, the up-regulated gene network appears to be more interconnected, suggesting a more complex and potentially more coordinated response. This may imply that the up-regulated genes are part of a robust activation process, possibly related to a stress response or other significant cellular changes in the combination of alcohol feeding and aging. Key up-regulated gene pathways in connecting aging with alcohol feeding include the cellular response to hormones, apoptosis, and cellular response to starvation.

Volcano plot analysis revealed distinct gene expression profiles among the experimental conditions (Figure 3E). Notably, genes such as *Plin4*, which is responsible for lipid droplet regulation, *Hif3a*, a transcription factor that regulates gene expression in response to low oxygen, and *Fmo2*, a flavin-containing monooxygenase, showed significant upregulation in the 23M CD group compared to the 3M CD group. These findings suggest that aging may increase lipid and xenobiotic metabolism and cause hypoxia in mouse brains. It was observed that both *Plin4* and *Pnpla2* genes showed higher expression in the 3M ED group compared to the 3M CD group. However, among the 23M ED and 23M CD groups, only *Pnpla2* was found to have higher expression, while *Plin4* did not show a significant difference. This suggests that alcohol may cause general lipid metabolism changes regardless of age. Notably, *Fkbp5* (FK506-binding protein 5) was identified as the highest expression gene in both the 23M ED and 3M ED groups but not in the 23M CD group. This suggests that *Fkbp5* may be specifically induced by alcohol. FKBP5 is a key molecule in the stress response and a particular single nucleotide polymorphism (SNP) has been implicated in the hypothalamic-pituitary-adrenal (HPA) axis and the development of stress-related psychiatric disorders such as posttraumatic stress disorder (PTSD), as well as the pathophysiology of stress-related disorders and AD [40,41].

**Figure 4:**
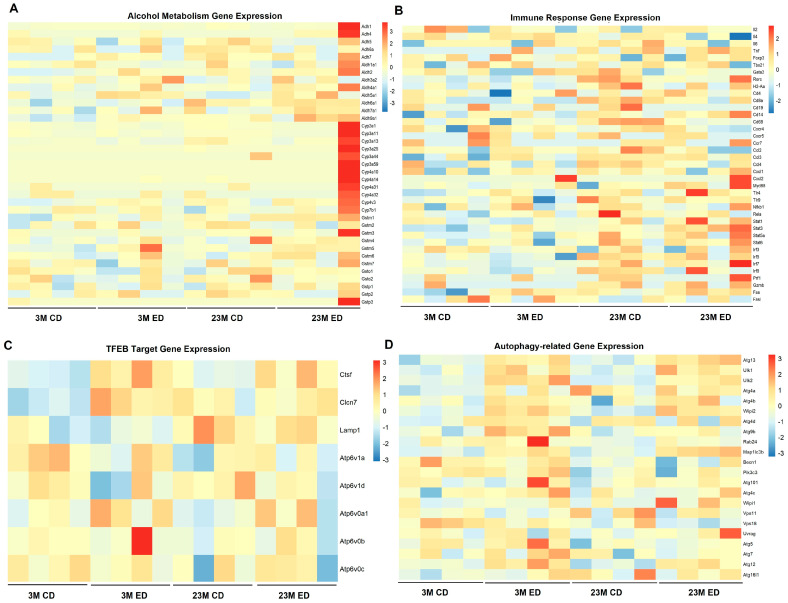
Heatmap analysis from the RNAseq dataset of Gao-binge alcohol-fed young and aged mouse hippocampi. Male 3-month-old young mice (3M) and 23-month-old aged mice (23M) were subjected to Gao-binge alcohol feeding. mRNAs were extracted from the mouse hippocampi after the feeding and subjected to RNAseq analysis. The color scale represents the magnitude of gene expression, with warmer colors (red) indicating higher expression and cooler colors (blue) indicating lower expression. The x-axis represents different experimental conditions, while the y-axis corresponds to the genes of interest in different pathways. The heatmap was generated using R with the ggplot2 package (version 3.5.1). (**A**) Heatmap analysis of alcohol metabolism gene expression from the RNAseq dataset. (**B**) Heatmap analysis of immune response gene expression from the RNAseq dataset. Heatmap analysis of TFEB target genes (**C**) and autophagy genes (**D**) from the RNAseq dataset.

A heatmap analysis of the RNAseq data showed that alcohol metabolism genes such as *Ahd, Aldh*, and *Cyp2e1* increased in alcohol-fed mice regardless of age (Figure 4A), suggesting the brain may also be able to metabolize alcohol partially. On the other hand, the expression of inflammatory genes was generally higher in aged mice compared to young mice, regardless of alcohol consumption (Figure 4B). We previously showed that alcohol-feeding increased mtDNA release and activation of cGAS-STING-mediated innate immune response [36]. Interestingly, the expression levels of *Irf3, Irf5, Ifr7,* and *Irf8* increased markedly in aging and alcohol-fed aged mice, suggesting aging may also promote mtDNA release and innate immune response in the brain. Additionally, some of the TFEB target genes including *Atpv1a*, *Atpv0c*, *Atpv0b*, *Atpv1h*, and autophagy-related genes (*Atg4b*, *Atg12*, *Wipi2*, *Atg4c*, *Atg4d*, *Pik3c3*, and *Vps18*) were downregulated in aged mice as compared to young mice, with alcohol consumption having little effect (Figure 4C,D), indicating that aging may impair TFEB-mediated autophagy in the mouse brain.

### 3.3. Chronic Alcohol Feeding Increases Cytosolic Retention of TFEB in Young and Aged Mouse Brains but Does Not Significantly Affect the Levels of Phosphorylated Tau and Histology Changes in Mouse Brains

**Figure 5:**
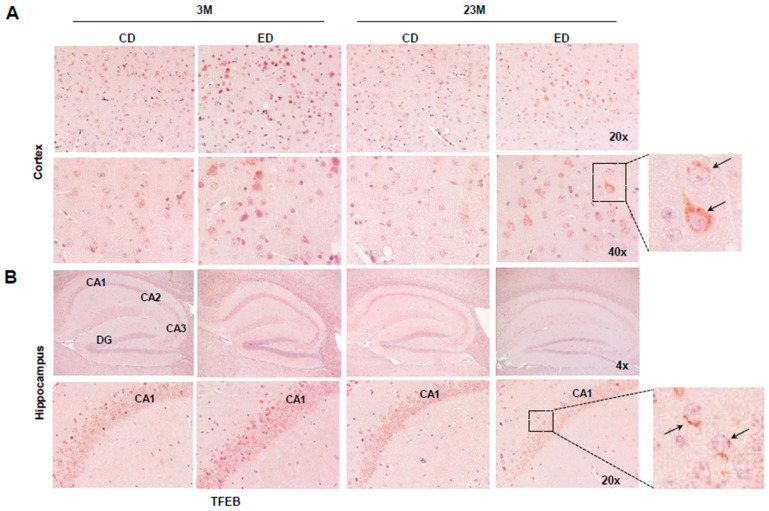
Immunohistochemistry staining of cortices and hippocampi TFEB in chronic EtOH-fed young mice and aged mice. Male 3-month-old young mice (3M) and 23-month-old aged mice (23M) were subjected to chronic EtOH feeding for 4 weeks. (**A**) Representative images of TFEB IHC staining of mouse cortices and (**B**) hippocampi are shown. Arrows denote cytosolic TFEB staining. CA: Cornu Ammonis, DG: dentate gyrus.

**Figure 6:**
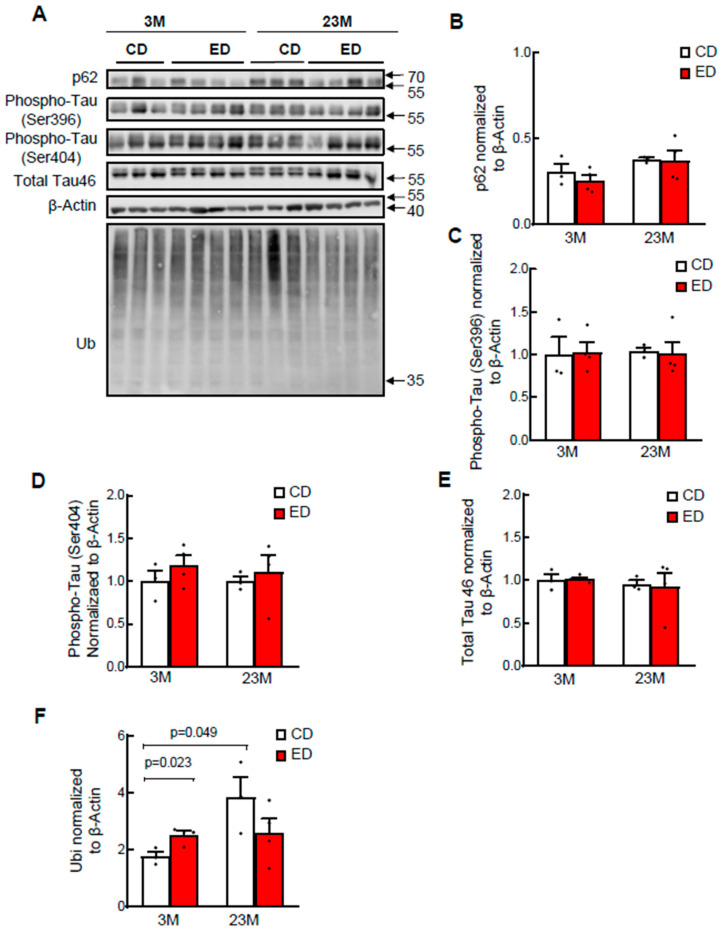
Chronic EtOH feeding does not affect Tau levels but increases levels of protein ubiquitination in young but not in aged mouse brains. Male 3-month-old young mice and 23-month-old aged mice were subjected to chronic EtOH feeding for 4 weeks. (**A**) Cortex brain lysates were subjected to western blot analysis followed by (**B**–**F**) Densitometry analysis of (**A**), which are normalized to loading control β-actin. Data are presented as mean ± SEM (n = 3–4).

**Figure 7:**
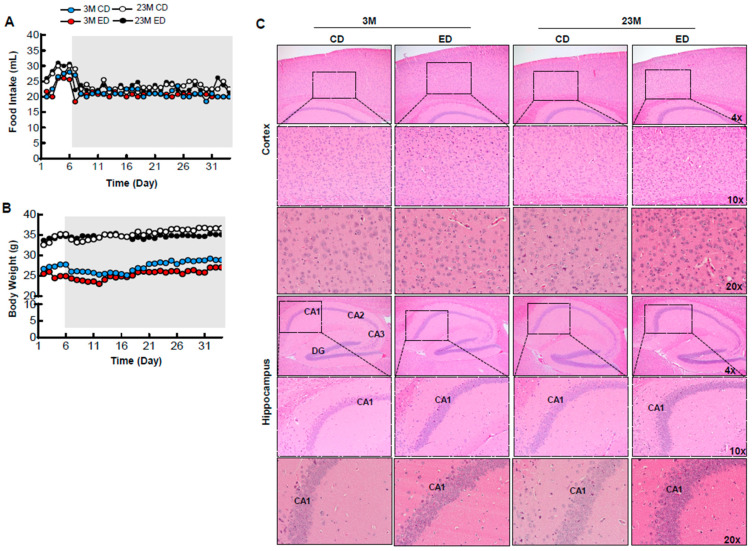
Chronic EtOH feeding increases apoptotic-like cell populations in young mice but not in aged mice brains, and without affecting body weight and food intake. Male 3-month-old young mice and 23-month-old aged mice were subjected to Gao-binge alcohol feeding. (**A**) Food intake, and (**B**) body weight (BW) were measured (n = 4–6). (**C**) Representative brain hematoxylin and eosin images are shown. Lower panels are enlarged images from the boxed areas. Original magnifications (4× and 10×).

We next determined whether chronic ethanol feeding alone (EtOH) for 4 weeks would affect brain TFEB and AD-related markers in young and aged mice. IHC staining for TFEB revealed increased cytosolic TFEB in alcohol-fed young and aged mouse cortex, but only increased in alcohol-fed aged mouse hippocampi concurrent with decreased nuclear staining (Figure 5A,B). Results from the western blot analysis showed that chronic alcohol feeding alone had little effect on levels of phosphorylated Tau and p62 in both young and aged mouse hippocampi (Figure 6A–E). The levels of ubiquitinated proteins were higher in aged mouse hippocampi compared with young mice fed the control diet. However, chronic alcohol feeding increased levels of ubiquitinated proteins in young but not aged mouse hippocampi (Figure 6A,F). Both young and aged mice consumed similar levels of ethanol during the 4-week alcohol feeding (Figure 7A). Alcohol feeding decreased weight gain in young but not aged mice (Figure 7B). Moreover, H&E staining showed no obvious changes in brain histology in both young and aged mice regardless of alcohol feeding (Figure 7C). Together, these data suggest that chronic alcohol feeding increases cytosolic TFEB retention but does not impact brain histology or AD-related markers in young and aged mice.

### 3.4. Aging Increases Synaptic Loss

**Figure 8:**
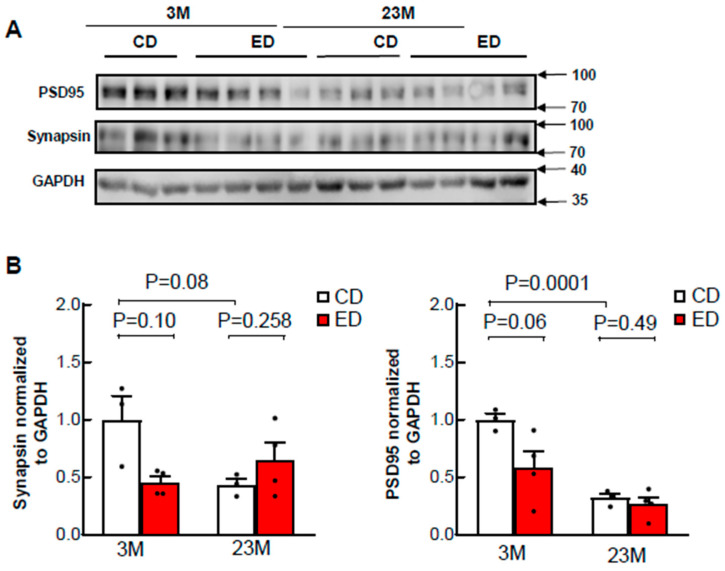
Chronic ethanol feeding has no effects on synapses but synapses are reduced in aged mice compared to young mice. Male 3-month-old young mice (3M) and 23-month-old aged mice (23M) were subjected to chronic EtOH feeding for 4 weeks. (**A**) Cortex brain lysates were subjected to western blot analysis followed by (**B**) Densitometry analysis of (**A**), which are normalized to loading control GAPDH. Data are presented as mean ± SEM (n = 3–4) in (**B**). *p*-value; one-way analysis of variance with Bonferroni’s post hoc test.

Synaptic loss and neurofibrillary pathology in the limbic system and neocortex contribute to AD cognitive decline [42]. A marked loss of the presynaptic markers synapsin-1 and synaptophysin, as well as the postsynaptic marker PSD-95, is observed in individuals with mild cognitive impairment and is associated with both prodromal and advanced stages of AD [43,44]. Chronic alcohol feeding significantly reduced levels of hippocampal PSD95 and synapsin. Additionally, PSD95 and synapsin were lower in aged mice (23 months old) than they were in young mice (3 months old). Interestingly, chronic alcohol consumption did not affect the levels of these proteins in the hippocampi of the aged mice (Figure 8A,B).

### 3.5. Aging Affects Spatial Memory to a Greater Extent than Alcohol

**Figure 9:**
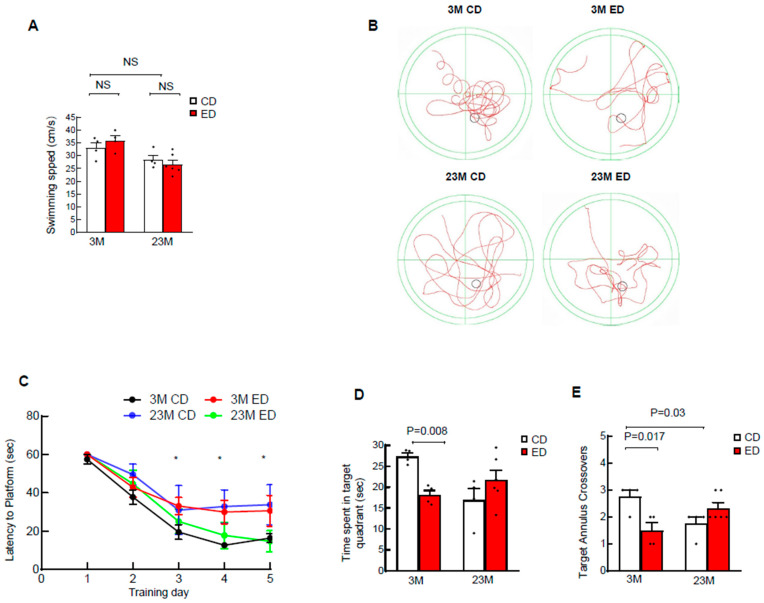
Chronic EtOH feeding impairs spatial learning and memory of young mice but not aged mice in the Morris Water Maze test. Male 3-month-old young mice (3M) and 23-month-old aged mice (23M) were subjected to chronic EtOH feeding for 4 weeks, followed by a Morris Water Maze test. (**A**) Swimming speed of indicated four mouse groups. (**B**) Representative images of swimming paths of indicated four mice groups during the probe test. (**C**) Escape latency decreased across the training days, representing the spatial learning ability of four groups of young and aged mice fed with or without EtOH. 3M control vs. 23M control. * p < 0.05*;* one-way analysis of variance with Bonferroni’s post hoc test. (**D**) Spatial reference memory of indicated four mice groups evaluated by time spent in the target quadrant and (**E**) number of times crossing the target platform. Data are presented as mean ± SEM. 3-month-old mice: Control, *n =* 4; EtOH, *n =* 4; 23-month-old mice: Control, *n =* 4; EtOH, *n =* 6; one-way analysis of variance with Bonferroni’s post hoc test.

The Morris Water Maze test assesses reference and working memory acquisition and retention [33,34]. Spatial memory acquisition was demonstrated by a decreased latency in finding the escape platform and an increased efficiency in reaching the platform. Neither aging nor alcohol feeding affected the swimming speed (Figure 9A), suggesting motor neurons and locomotive functions of the mice were not compromised by either advanced age or alcohol feeding. Swimming path analysis and quantification of the latency to platform time showed that aged mice fed the control diet had increased latency to find the platform compared with young mice fed the control diet (Figure 9B,C). Aged mice fed the control diet also showed a trend of reduced time spent in the target quadrant (Figure 9D), and significantly fewer target annulus crossovers (Figure 9E) compared with young mice fed the control diet. Interestingly, alcohol-fed young but not aged mice had increased latency to the platform and decreased time spent in target and numbers of target annulus crossovers compared to their respective control diet-fed mice (Figure 9C–E).

**Figure 10:**
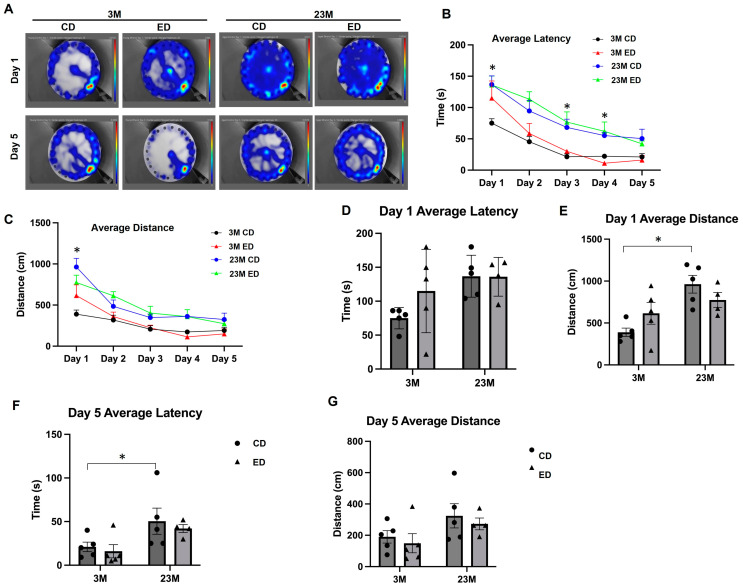
Aging but not chronic EtOH feeding impairs mouse spatial learning and memory in the Barnes Maze test. Male 3-month-old young mice (3M) and 23-month-old aged mice (23M) were subjected to chronic EtOH feeding for 4 weeks, followed by Barnes Maze testing. (**A**) Representative heat maps of Days 1 and 5 from each treatment group. (**B**) The average latency to escape for each test day. (**C**) Average distance traveled to escape for young and aged mice fed with and without EtOH. (**D**) Day 1 average latency to escape for 3-month-old and 23-month-old mice fed with or without EtOH. 3M control vs. 23M control (**B**,**C**). * *p* < 0.05; one-way analysis of variance with Dunn’s post hoc test. (**E**) Day 1 average distance traveled to escape for 3-month-old and 23-month-old mice fed with or without EtOH. (**F**) Day 5 average latency to escape for 3-month-old and 23-month-old mice fed with or without EtOH. (**G**) Day 5 average distance traveled to escape for 3-month-old and 23-month-old mice fed with or without EtOH. Data are presented as mean ± SEM. 3-month-old: CD, n = 5; ED, n = 5; 23-month-old: CD, n = 5; ED, n = 4. * *p* < 0.05; one-way analysis of variance with Dunn’s post hoc test.

The Barnes Maze test is a dry-land-based behavioral test that is used to study rodent spatial memory [35]. From a behavior interrogation perspective, it is similar to the Morris Water Maze but much less stressful to the rodents, who intensely dislike immersion in water. Heat map analysis of movement and quantification of the latency to escape the maze revealed that aged mice fed the control diet had increased latency of time to escape compared with young mice fed the control diet (Figure 10A,B), which is consistent with the Morris Water Maze test results, supporting the conclusion that aging impairs mouse spatial memory acquisition. Aged mice also showed an increased average distance to the goal compared with the young mice, which was not affected by alcohol feeding (Figure 10A,G). Alcohol-fed young mice had no significant effects on latency to goal and distance to escape when compared to the control diet-fed young mice (Figure 10D–G). Together, these data indicate that aging is associated with larger decrements in spatial memory performance than are the effects of alcohol.

## 4. Discussion

In this study, we used two alcohol-feeding models to address the impact of alcohol and aging on brain autophagy and TFEB as well as mouse memory acquisition. We used the Gao-binge alcohol model, which is widely used to mimic chronic drinking plus binge alcohol consumption [45]. Biochemical, transcriptomic, and histologic analysis of hippocampi from young and aged mice showed impaired TFEB-mediated autophagy in Gao-binge alcohol-fed young mice. Brains from aged mice showed defective TFEB-mediated autophagy relative to brains from young mice, and Gao-binge alcohol feeding did not exacerbate the age-related changes. Aged mice had impaired spatial memory acquisition in both the Morris Water Maze and Barnes Maze tests. While alcohol feeding slightly impaired spatial memory acquisition in young mice, alcohol had little effect or even slightly improved memory performance in aged mice.

AD is a neurodegenerative condition that is primarily associated with the abnormal processing and polymerization of normally soluble proteins. Gene mutations and aging can promote protein misfolding and aggregation, which is associated with neuron dysfunction and loss. AD is generally characterized by extracellular aggregates of Aβ plaques and intracellular aggregations of neurofibrillary tangles (NFTs) composed of hyperphosphorylated tau protein. In addition to Aβ plaques and tau aggregation, mitochondrial dysfunction is also associated with the disease [46]. Increasing evidence suggests impaired autophagy may cause an accumulation of Aβ and tau aggregates and contribute to the development of AD [24]. Numerous studies show autophagy is disturbed in AD patients, and potentially drives an accumulation of autophagic vehicles (AVs) within neurons [47,48]. Lysosomal biogenesis is regulated by TFEB. Dysfunctional TFEB has been linked to AD in both humans and experimental AD models [24,26]. Moreover, in AD transgenic mouse models, pharmacological activation or genetic overexpression of TFEB initiates a lysosomal biogenesis that clears Aβ and tau aggregates [29,30,32]. Aging is a well-known AD risk factor, and aging is associated with reductions in autophagy and TFEB [49,50].

We found aging alone slightly decreased brain TFEB levels with increased LC3-II, p62, and ubiquitination by western blot and IHC analysis, and alcohol feeding did not enhance these changes. Moreover, our transcriptome analysis also showed decreased expression of some TFEB target genes and autophagy genes in aged mouse brains, and alcohol exposure did not exacerbate these changes. This suggests aging more robustly reduces brain TFEB-mediated autophagy than alcohol.

Morris Water and Barnes Maze testing revealed that spatial memory in aged mice was impaired relative to young mice. Alcohol impaired spatial memory in Morris Water Maze but not Barnes Maze testing in young mice. Surprisingly, alcohol feeding did not worsen the age-related spatial memory decrements in aged mice. Our biochemical, transcriptome, and histological observations are generally in line with the results from the two spatial memory tests. Aged mice already have lower levels of TFEB in their brains, regardless of whether they consumed alcohol or not, while alcohol decreased TFEB in younger mice. Our findings support the notion that age-related reductions in autophagy and TFEB could contribute to the development of AD. C1, a curcumin analog, efficiently activated TFEB, enhanced autophagy and lysosomal activity, and reduced β-amyloid peptides and Tau aggregates in these models, which was accompanied by improved synaptic and cognitive function [32]. Further studies are needed to address whether TFEB is a valid therapeutic target [51].

Several possibilities could account for why alcohol did not worsen cognitive performance in aged mice. The duration of exposure was only 4 weeks. A more chronic treatment may have produced a different result. During the treatment period, we observed that the alcohol-fed mice increased their brain expression of alcohol metabolism-related genes. This suggests they mounted an acute adaptive response. We do not know whether this adaptive response persists under conditions of chronic alcohol exposure. Cognitive decline in humans is influenced by a variety of complex factors beyond aging and alcohol consumption. These factors include numerous socio-cultural mediators and moderators related to psychological variables and cognition [52]. Psychological stress, including neuroticism, stressful life events, and perceived stress, can increase the risk of developing dementia [53]. Moreover, having a mental disorder can lead to cognitive decline, and individuals who consume alcohol are more prone to developing mental disorders. Conversely, people with severe mental illnesses are also more likely to increase their alcohol intake. People with dementia may drink more alcohol because they may not remember how much they have consumed, although more research is needed to provide definitive evidence. Nonetheless, it is challenging to find a single animal model that accurately reflects the intricate relationship between alcohol consumption, psychological factors, and cognitive decline associated with conditions like AD.

## 5. Conclusions

The impact of alcohol consumption on AD risk remains unclear. Inconsistent results from past studies may reflect inter-study differences in how consumption amounts and patterns were defined [7,54]. Some studies indicate that moderate alcohol consumption may reduce AD risk in older populations [55,56]. Results from our alcohol animal models suggest that aging but not alcohol consumption is a more critical driver of spatial memory impairment and reduced brain autophagy flux. Given the limitations of the animal models, it is clear that additional research is needed to clarify the impact of alcohol on AD risk and pathogenesis in the future.

## Data Availability

The data presented in this study are available in GSE 283201.

## Supplementary Materials

The following supporting information can be downloaded at: https://www.mdpi.com/article/10.3390/biom14121537/s1, Western Blotting Figures.

## Abbreviations

AD, Alzheimer’s disease; ADRD, AD and its related dementia; AVs, autophagic vehicles; CLEAR, coordinated lysosomal expression and regulation; CD: Control diet; CNS, *central nervous system;* ED: ethanol diet; *Fkbp5*, FK506-binding protein 5; HPA, hypothalamic-pituitary-adrenal; LD, lipid droplets; NFTs, neurofibrillary tangles; PTSD, posttraumatic stress disorder; SNP, single nucleotide polymorphism; TFEB, Transcription factor EB.
