# Supplementary figures and images for: Late-Life Alcohol Exposure Does Not Exacerbate Age-Dependent Reductions in Mouse Spatial Memory and Brain TFEB Activity
Source: Biomolecules. 2024 Nov 30;14(12):1537. doi: 10.3390/biom14121537 (PMC11673978; doi:10.3390/biom14121537)

Figure 1C

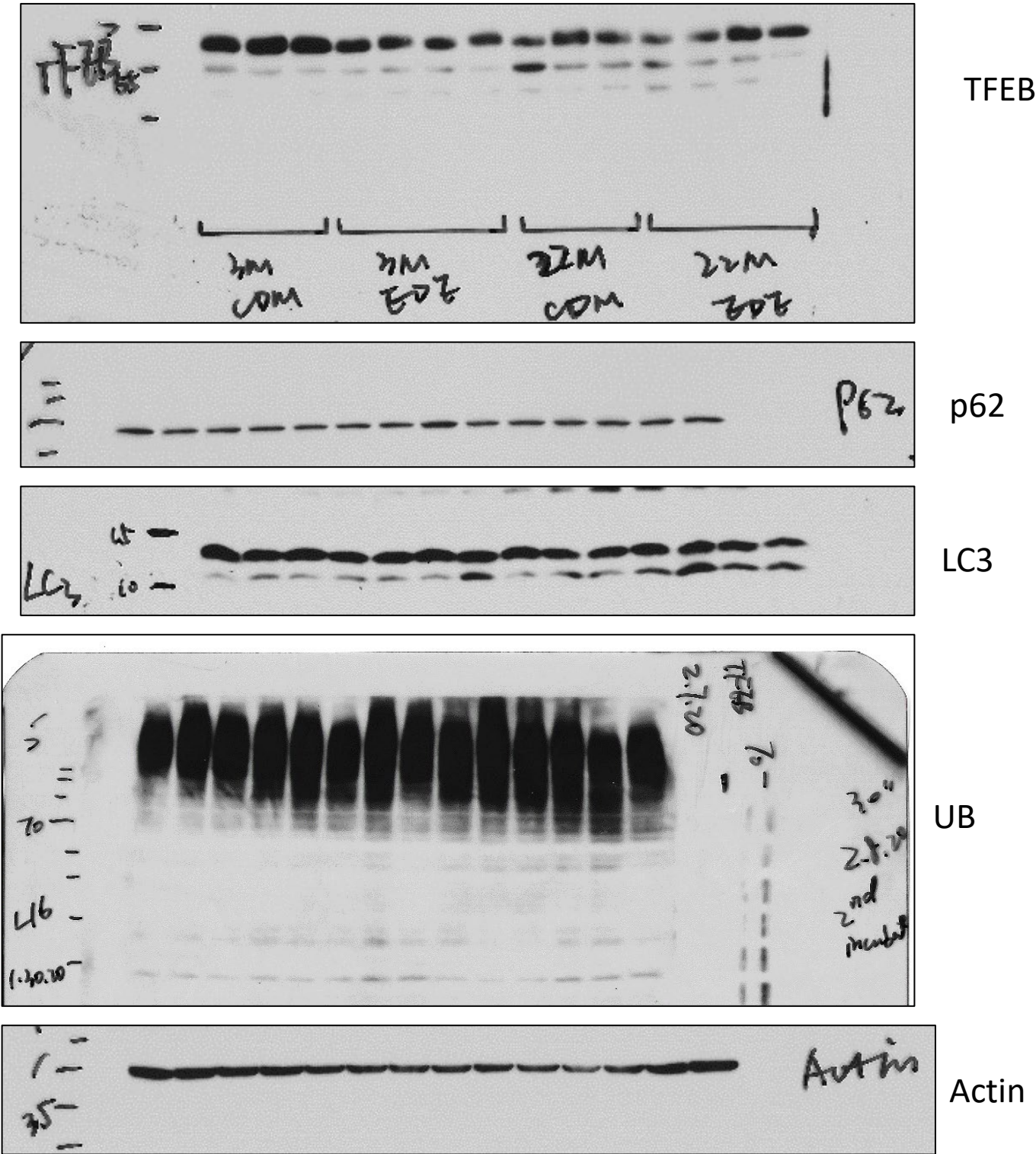

Figure 6A

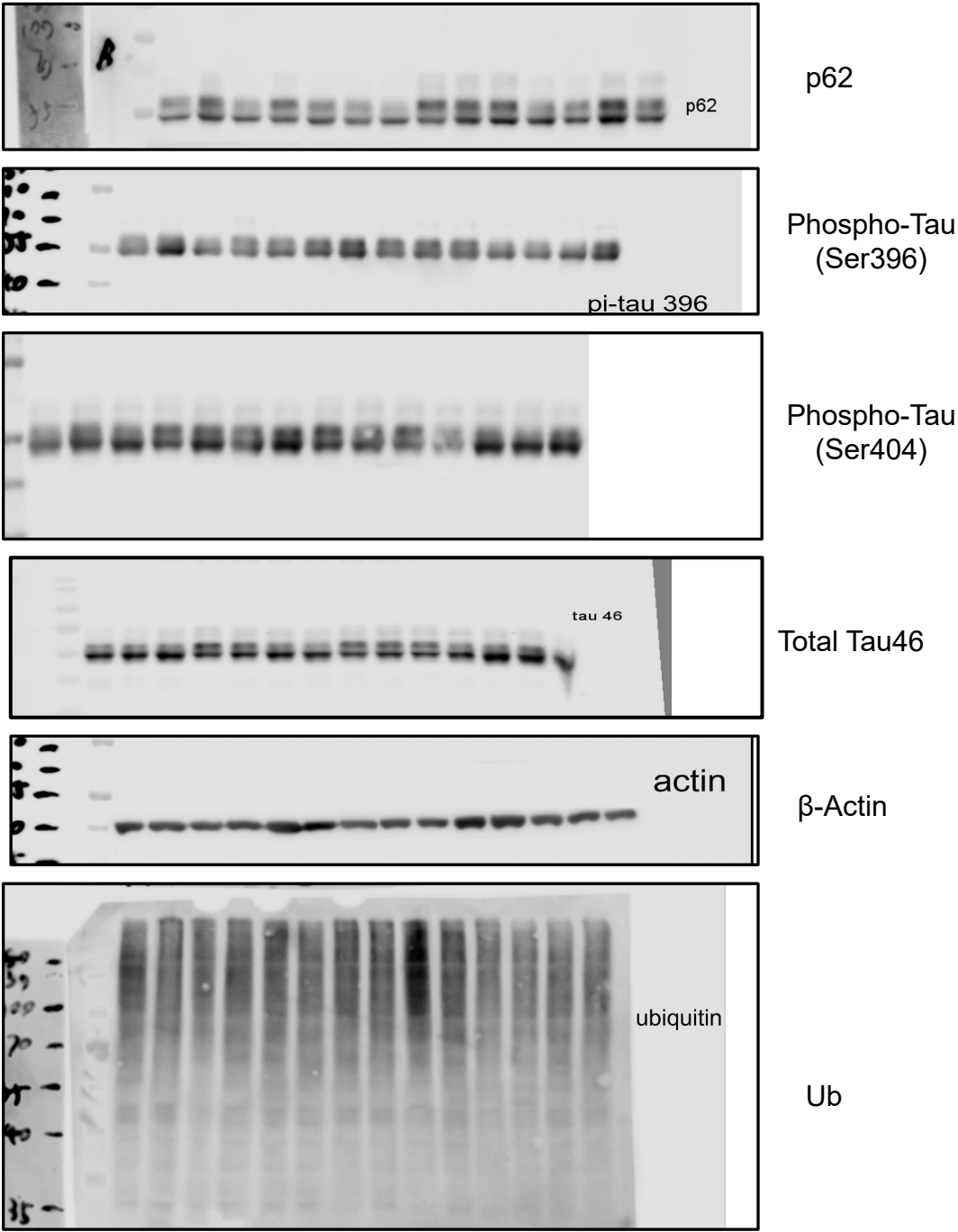

Figure 8

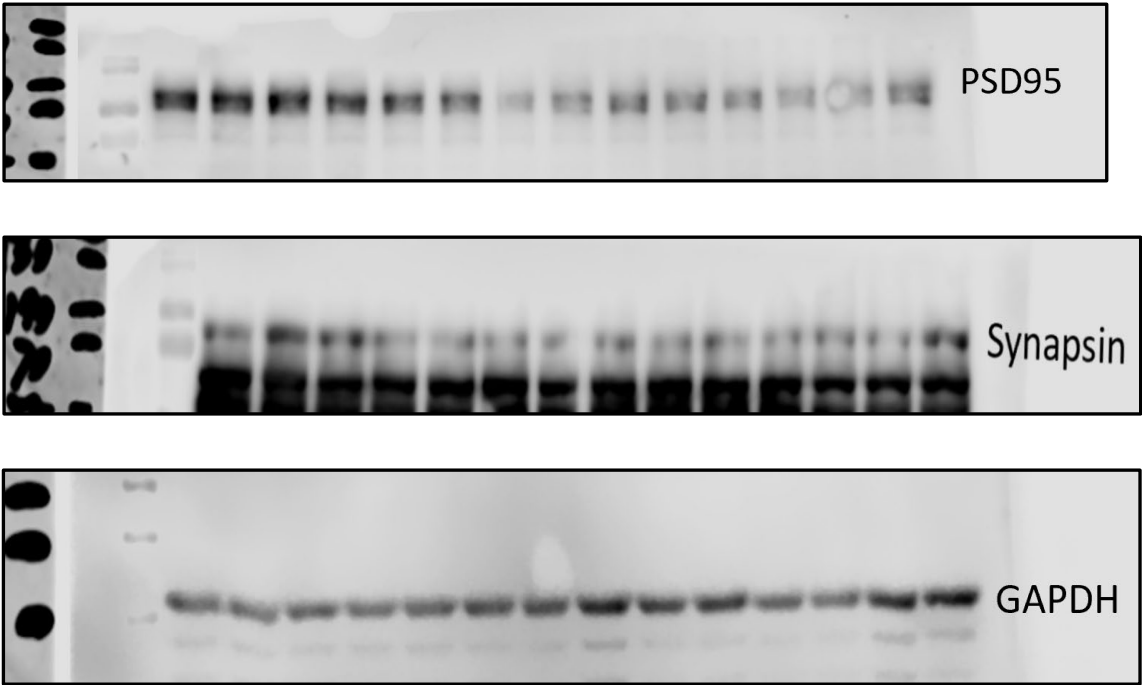

Supplement: Supplementary file 1 [file biomolecules-14-01537-s001.zip › biomolecules-3247578-supplementary.pdf]
